# Influence of a Planning Intervention on Physical Activity Behavior: the Moderating Role of Intentions and Executive Functions in a Randomized Controlled Trial

**DOI:** 10.1007/s12529-020-09864-x

**Published:** 2020-02-19

**Authors:** Ines Pfeffer, Tilo Strobach

**Affiliations:** Medical School Hamburg, Faculty of Human Science, University of Applied Sciences and Medical University, Am Kaiserkai 1, 20457 Hamburg, Germany

**Keywords:** Inhibition, Updating, Shifting, Exercise, Intention–behavior gap, Self-regulation

## Abstract

**Background:**

Planning and executive functions (EFs; inhibition, updating, shifting) are self-regulatory variables that help people to become and stay physically active. The aim of this study was to examine how and for whom a planning intervention affects physical activity (PA) behavior in the short term. Therefore, the mediating role of planning and the moderating role of intentions and EFs for the planning–behavior link were examined.

**Method:**

In a randomized control trial with two treatment groups (planning group vs. control group) and two points of measurement (t1 and t2, 1 week apart), *n* = 200 students participated in both measurements. At t1, participants filled in standardized questionnaires assessing PA behavior, intention, and planning. Computer-based tests assessed the following EFs: inhibition, updating, and shifting. At t2, planning and PA behavior were measured again. Moderated mediation analyses were conducted.

**Results:**

A significant increase in PA between t1 and t2 was found for the planning group compared with the control group. Furthermore, planning cognitions significantly mediated the effect of the planning group on behavior and intention, as well as the EF updating moderated the association between planning and behavior. Forming plans was particularly beneficial for participants with high intentions and lower updating performance.

**Conclusion:**

Planning enhances PA behavior, particularly when PA intention is high. Poor performance in updating can be compensated by planning since encouraging people to generate plans might facilitate automatic enactment of the behavior.

## Introduction

Regular physical activity (PA)^1^ can be predicted by intentions (i.e., motivation) and self-regulation [1, 2]. However, review studies have observed a substantial amount of unexplained variance in PA behavior when predicting this behavior from intentions [3, 4]. This discrepancy is termed the intention–behavior gap. Self-regulatory techniques (e.g., planning) as well as cognitive variables (e.g., executive functions (EFs)^2^) are self-regulatory factors that might close this gap [5, 6]. Planning interventions were repeatedly shown to enhance the PA level [7], and current research is directed towards detecting variables that might explain for whom and how a planning intervention successfully translates into PA behavior. Therefore, in this study, the moderating roles of intentions and EFs for translating plans into activity were investigated.

### The Role of Planning and Planning Interventions on Physical Activity Behavior

Theories such as the Health Action Process Approach (HAPA; [8]) differentiate between a motivational phase where intentions are formed and the volitional phase where the intended behavior is adopted and maintained [6, 8, 9]. Planning is proposed to be a volitional mechanism by which an intended goal is translated into action. Planning of a health behavior is based on the idea of implementation intentions [10], and it represents a prospective self-regulatory technique [11]. This technique refers to the link between a situation (a specific cue) and a goal-oriented response (e.g., “*If* situation X is encountered, *then* I will perform response Y”) by making the mechanisms that reduce the gap between goal intentions and goal attainment explicit [10]. Linking a given situation to a specific behavioral response will make the behavioral response more likely to occur when the situational cue is encountered [10, 12].

Planning PA behavior can comprise two types of plans: (a) action plans and (b) coping plans. Action plans [13] should include time-related cues (“when”), the complex external environment (“where”; [14]), and the specification of “how,” “with whom,” and “how long” the behavior should be performed. This approach is often complemented by the formation of coping plans. Coping plans include the anticipation of personal risk situations (i.e., situations that might erode the implementation of the action plan) and a detailed plan of how to cope with these obstacles [11]. Action plans are task-facilitating cognitions that help to enact a specific behavior, and they are thought to be particularly useful for the *adoption* of complex behaviors. In contrast, coping plans are distraction-inhibiting cognitions [11] that help to the pursuit of a goal intention even if obstacles arise, and this planning type is assumed to be more important for behavioral *maintenance* [15].

People who form plans are more likely to act in the intended way. There is evidence from several studies for the mediating role of action and coping planning cognitions, explaining the intention–behavior relationship [8, 15, 16]. During planning interventions, participants are usually asked to generate action plans (e.g., up to three plans) indicating when, where, how, with whom, and how long to perform the PA behavior. Based on these action plans, participants are encouraged to identify their individual obstacles and barriers that might impede the enactment of the action plans. Then, participants are encouraged to make coping plans indicating what to do if something interferes with the action plans [17, 18]. Meta-analyses have concluded that the overall effect of planning interventions for increasing PA is small to medium, with greater effects documented when coping planning complements action planning [7, 19, 20]. In a recent planning intervention study, Pfeffer and Strobach [21] showed that the effect of a planning intervention on PA behavior over 1 week was mediated by self-reported planning (i.e., the level of details in the participant’s plans) measured in a follow-up. However, having an intention for PA behavior and generating action as well as coping plans does not necessarily bridge the intention–behavior gap [7]. Hence, future research should identify relevant moderators and mediators of planning intervention effects [18] to better understand for whom and how such planning interventions work [18, 22]. To elaborate on the underlying causal mechanisms, a short timeframe between planning and behavior assessment seems to be suitable.

### Executive Functions and PA Behavior

There is increasing evidence suggesting that EFs subserve effective self-regulation (i.e., choosing and pursuing goals in a way that leads to goal attainment) of PA behavior [9, 23–26]. Furthermore, EFs are cognitive operations that subserve goal-directed processing and enable effortful top-down control of behavior over lower-level cognitive processes, such as unwanted habits or automatic impulses. In their unity/diversity framework, Miyake and colleagues [27, 28] systematized the different processes involving EFs by analyzing behavioral performance in EF tests. Miyake and Friedman [27, 28] primarily distinguished between three EF domains: *inhibition*, *updating*, and *shifting*. Inhibition refers to overriding dominant or prepotent responses, updating refers to monitoring and manipulating working memory contents, and shifting is associated with switching flexibly between different tasks or mental sets (i.e., cognitive flexibility). While the executive domains tap into some common variability (i.e., unity), they also show separability (i.e., diversity). On the one hand, this means that the correlations among the three EF latent variables are substantial and reflect similar underlying mechanisms. On the other hand, these correlations are far from perfect (i.e., 1.0), supported by the observation that the three EFs differentially relate to other measures, such as well-known neuropsychological tests of frontal lobe functioning [28] and IQ [29].

One main aspect of successful self-regulation in health behavior is the ability to actively inhibit or override behavioral responses that are incompatible with one’s goals [30]. Consequently, participants with low levels of inhibition are less successful at translating their intentions into action and have a bigger intention–behavior gap [5, 31, 32]. However, empirical studies examining whether the executive domains updating and shifting are associated with the gap between PA intention and behavior are scarce. Theory states that successful self-regulation entails the representation of goals (e.g., the intention to be physically active) and goal-relevant information (e.g., detailed action and coping plans; [30]). Updating might subserve this active mental representation of an individual’s goal and the associated means by which the goal that is recruited from long-term memory can be attained [30]. Indeed, several studies have shown that having a health-related goal (e.g., the intention to be physically active) may only be beneficial when an individual has sufficient updating ability [33–36]. Furthermore, high shifting ability might facilitate pursuit of a goal by allowing individuals to abandon suboptimal means (e.g., inappropriate plans) and to pursue alternative means to reach an intended goal, such as when barriers occur (means-shifting; [30, 37]). Accordingly, it has been shown that superior performance in shifting is associated with a lower intention–behavior gap in the domain of healthy eating and with more flexible self-regulatory techniques, leading to higher PA levels [31, 38]. The results of these studies indicate that EFs represent considerable abilities for successful self-regulation of health behaviors. However, the roles of EFs in self-regulatory intervention studies aiming to bridge the intention–behavior gap have been rarely examined.

### The Role of EFs in Planning Interventions

Making plans by mentally linking a situational cue with a behavioral response will lead to an automatic elicitation of this behavior in case the situational cue is encountered [10]. The automatic nature of the behavior achieved by generating plans could help people with low inhibition, updating, and shifting abilities to act more in line with their intentions and plans. Automatic behaviors are less susceptible to distraction by unwanted impulses that might impede goal attainment [30]. Furthermore, a mental representation of the goal intention and goal shielding subserved by the updating function is not necessarily needed anymore. Planning could also prevent shifting the goal away from the intended behavior towards tempting alternatives. When the initial action plan does not work (e.g., because of changes in the environment), people find themselves in a difficult situation where self-regulatory failure is likely and elaborated self-regulation based on EF abilities is needed. Forming coping plans might support people with low EF abilities to still act in the intended way since the alternative plans were generated in advance and are elicited automatically when the initial action plan fails. Effortful inhibition of the impulse to watch television instead of being physically active, updating of the goal intention, or purposefully shifting attention away from the initial plan to an alternative means of goal attainment is almost not necessary. In summary, people with lower EF abilities might benefit from action and coping planning since behavioral control is transferred to the situation [10].

Only a few studies have examined the moderating role of EFs in planning intervention studies of health behavior in young adults. The results of two previous studies suggest that planning interventions are a compensating technique for people with lower EF abilities [35, 39]. Hall and colleagues [39] found that generating action and coping plans compensated for poor performance in an inhibition task (go/no-go task) with regard to the intention–behavior gap in the context of PA behavior. Allan et al. [35] found comparable results for behavior related to the intake of snacks using a planning skill measure (tower task). However, in this intervention study, snacking behavior was not used as a dependent variable (study 2 in [35]) but instead, the completion of a food diary, which is an indirect indicator of snacking behavior itself, was used. Furthermore, both intervention studies did not examine the mediating role of plans measured in a follow-up with standardized planning scales. Consequently, a moderated mediation model examining the moderating role of intentions and EF performance for the mediation effect of planning on PA behavior in a planning intervention study has not been tested.

An additional limitation of previous studies was that these studies measured EFs with only one test (go/no-go or tower task). Thus, these studies’ measurements are not based on an elaborated model of EFs, as proposed by Miyake and colleagues [27, 28]. Furthermore, they ignore the task impurity problem, namely that any target EF must be embedded within a specific task so that the target EF has something to operate on [27, 40]. Any score derived from an EF task necessarily includes systematic variance attributable to non-EF processes associated with that specific task (e.g., perceptual processes, response processes, and general processing speed). Unfortunately, this non-EF variance is substantial, making it difficult to plainly measure the target EF variance. To alleviate this task-impurity problem, a latent variable approach is required. In this approach, one selects multiple tasks that seem different on the surface but still tap into the target EF. If tasks are chosen such that they share little non-EF variance, one can statistically extract what is common across those tasks and use the resulting “purer” latent variable as the measure of the EF. The contribution of such a strategy allows for investigation of the moderating role of EFs on a planning intervention, not on a task level but on a latent factor level.

### The Present Study

**Fig. 1:**
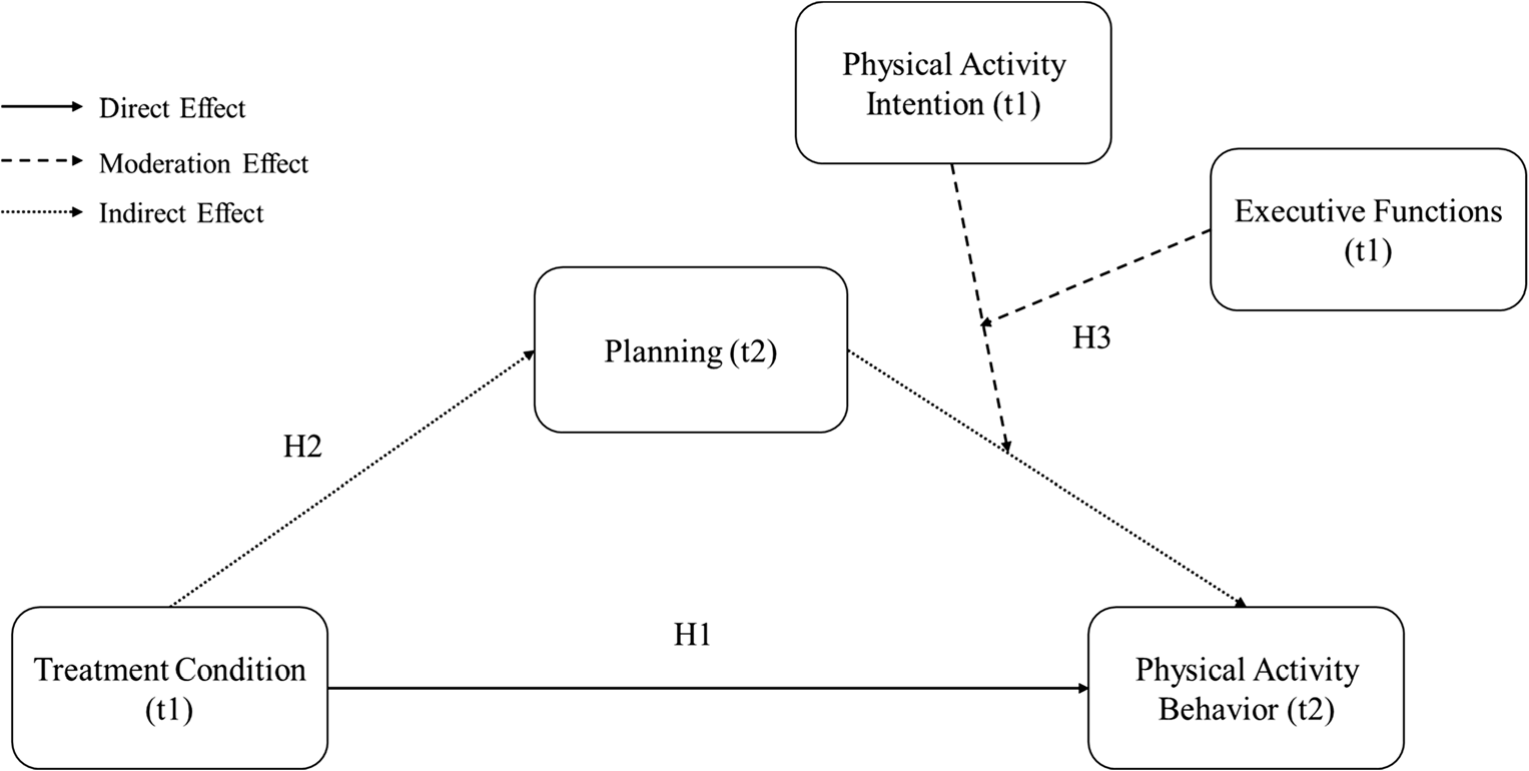
Conceptual moderated mediation model underlying the current study examining the effects of a planning intervention on physical activity behavior. t1, first assessment; t2, second assessment

In the present study, it was assumed that PA behavior will be influenced by the treatment condition (planning intervention vs. control intervention). Previous research was extended by assessing planning as a mediator between the planning intervention and behavior. Furthermore, it was assumed that intentions and EFs proposed by Miyake and Friedman [27, 28] would moderate the association between planning and behavior. Two tests per EF domain were performed, which might allow inferences to be made at the latent level of each domain (i.e., generalized across tests) rather than on only a single test. In line with that, moderated mediation effects, as depicted in Fig. 1, were expected.

It was hypothesized that (1) the treatment condition would influence planning and PA behavior. Participants in the planning condition will have more detailed plans (i.e., higher scores on the planning measure) and will be more physically active at t2 compared with participants in the control condition. Since intention is a necessary prerequisite of successful plans, it was further assumed that (2) intention and EFs would moderate the influence of planning on PA behavior. The influence of planning on PA will be stronger for participants with higher intentions and lower EF abilities (compensating effect of plans) compared with individuals with lower intentions and superior performance in EFs. In line with that, (3) planning will be a stronger mediator between the treatment group and PA behavior for participants with higher intentions and poorer EFs compared with participants with lower intentions and superior performance in EFs.

## Methods

### Study Design

A randomized controlled trial with two groups (a planning group and a control group) and two laboratory assessments, with a 1-week interval between the 2 assessments, was conducted.

#### Procedure

Participants were contacted and tested by trained assistants. The procedure was in accordance with common ethical standards and approved by the institutional research committee (MSH-18/39). Data were obtained using an online survey tool for quantitative research (Software Unipark QuestBack EFS Survey 10.8 for academic research; Cologne, Germany) and the software package *Presentation* (version 18.1). After providing informed consent, sociodemographic data and control variables (i.e., age, sex, past PA behavior, action, and coping planning), as well as the moderators (i.e., PA intention and EFs), were assessed at t1. After these assessments, participants were assigned by a computer tool (research randomizer; [41]) at random (block randomization) to 1 of 2 groups (planning group vs. control group) and received the allocated intervention. Action and coping planning (mediators), as well as PA behavior (dependent variable), were again assessed 1 week later at t2. The first assessment took about 2.0 h, and the second assessment took about 5 min.

#### Treatment Conditions

After completing t1 assessments, participants of the *planning group* received a supervised planning intervention at the end of t1, which took about 15 min. Participants were asked to indicate what kinds of PA (with at least moderate intensity, that is, sweating and breathing harder) they would like to execute for increasing their PA level. They were encouraged to write up to 3 activities on a planning sheet. Then, the participants were told how the action plans should be structured (“when,” “where,” “with whom,” “how,” and “how long” to perform the PA) and were invited to write up to 3 PA action plans for the next 7 days. Subsequently, participants were informed that barriers may impede these action plans, and they were encouraged to identify up to 3 individual risk situations that might interfere with the execution of the action plans. Finally, participants were asked to write up to 3 coping plans to overcome these individual obstacles [42].

Participants of the *control group* read a text from a popular scientific journal without reference to PA behavior in the presence of the experimenter for about 15 min [21].

### Participants

Sample size estimation should be based on a comparable effect size from previous studies. Since none of the previous studies conducted moderated mediation analyses and tested the interaction effect planning × intention × EF, options for a priori sample size estimation were limited. However, to detect a small effect size (*f*^*2*^ = 0.05) for the interaction effect planning × intention × EF, a minimal a priori sample size of *n* = 160 participants was calculated for linear multiple regression analyses (*R*^*2*^ increase), given a statistical power of (1 − β) = 0.80 and a level of significance of α = 0.05 and testing 1 predictor (three-way interaction term) by including up to 8 predictors (including 4 control variables; see below for details about predictors) in the model [43].

Two hundred seven undergraduate and graduate students voluntarily participated in the study or in exchange for course credit in the first assessment (t1), of which *n* = 200 also completed the second assessment (t2). Data of 6 and 2 participants were eliminated due to extreme values in PA behavior at t1 and t2, respectively. The performance value in a shifting paradigm (i.e., the alternating runs paradigm) of one participant was eliminated because of an extreme value. The remaining tests/scores and participants showed no such outliers. Thus, the final sample consisted of *n* = 191 participants (137 females, 54 males; age *M* = 22.70 years; SD = 2.53; range 18–34; *n* = 102 in the planning condition and *n* = 89 in the control condition). Missing values were found in the following EF measures due to technical problems during data recording: *n* = 4 in the go/no-go task, *n* = 1 in the stop-signal task, *n* = 9 in the N-back task, *n* = 6 in the visual memory task (updating), *n* = 2 in the alternating runs paradigm, and *n* = 2 in the task-cueing paradigm.

### Measures

#### PA Intention and Behavior

Since our study is based on the work of Hall et al. [5, 39] and in order to achieve comparability with the results of Hall et al. [39], PA intention and behavior were measured in the same way as in the studies by Hall et al. To be able to control for the effects of *past PA behavior*, participants were asked to indicate the number of hours (to the nearest half hour) they engaged in vigorous physical activities during the *last 7 days* by using examples of behavioral criteria for vigorous intensity activities (e.g., running, jogging, soccer, vigorous swimming, and cycling) at t1. Vigorous PA was chosen following Hall et al. [5]. The authors found that this measure was significantly more reliable than moderate and light activity [44]. It was derived from the Stanford 7-day recall [45] and correlated *r* = 0.60 (*p* < 0.001) with tri-axial accelerometer-assessed PA in healthy young adults [5]. In addition, this measure has proven sensitive to behavioral intervention effects [46]. Also, other researchers have shown that single-item measures of PA performed as well as other short PA assessments regarding reliability and concurrent validity [47, 48].

PA intention was assessed in t1 by changing the temporal perspective of the self-report PA measure from past behavior to future behavior. Participants were asked to indicate the number of hours (to the nearest half hour) they intend to engage in vigorous PA during the next 7 days by using examples of behavioral criteria for vigorous intensity activities [5].

PA behavior, as a dependent variable, was assessed 1 week later at t2. For this purpose, the same measure as for past PA behavior was used. Participants were asked to indicate their PA behavior by assessing the number of hours (to the nearest half hour) they were engaged in vigorous physical activities during the last 7 days.

#### Action and Coping Planning (Mediators)

Action and coping planning were assessed with scales by Sniehotta et al. [11] at t1 and t2. Responses were made on four-point Likert scales ranging from 1 (not at all true) to 4 (absolutely true). Action planning was introduced by the stem “I have made detailed plans regarding…,” which was followed by 6 different items (e.g., “... when to do my physical activity”). The mean value of the 6 items was calculated, and higher scores represented more specific action plans. The coping planning scale was introduced by the words “I have made detailed plans regarding...,” followed by 4 items (e.g., “...what to do if something intervenes”). A mean value for coping planning was calculated from the 4 items, and the higher the score, the more specific the individual’s plans in terms of how to cope with difficulties and how to overcome obstacles [21]. The analyses revealed good (Cronbach’s alpha; action planning, α = 0.88; coping planning, α = 0.84) to very good (total planning score, α = 0.91) internal consistencies for both scales. Since action and coping planning were highly correlated in our study (*r* = 0.63), a total planning score was calculated by building the mean value of the action planning and coping planning mean scores to prevent multicollinearity in the model. Cronbach’s alpha for this scale was α = 0.88.

#### Tests on EFs

The following EF tests were performed: go/no-go (inhibition; [5]), stop-signal task (inhibition; [49]), N-back task (updating), visual memory task (updating; [50]), task-cueing paradigm (shifting; [51]), and alternating runs paradigm (shifting; [52]). The tests were described in detail in the study by Pfeffer and Strobach [9]. Performance scores in the go/no-go and the stop-signal tasks are illustrated by the reaction time in milliseconds. Therefore, the shorter this reaction time (i.e., the lower the performance scores), the higher the inhibition performance. Similarly, performance scores in the shifting tests are illustrated by the time (in milliseconds) to switch between different tasks (i.e., task-switching costs). The lower these costs (i.e., the lower performance scores), the higher the shifting performance. This relation is reversed for the updating tasks: the more information (i.e., the more items processed) can be held and updated in working memory (i.e., the higher the performance scores), the higher the updating performance. For half of the participants, the presentation order of the tests was as listed above, while the other half of participants performed these tasks in the reversed order. Stimulus presentation as well as reaction time (RT) and correct response measurements in all of these EF tests was performed on a Windows-compatible PC. Participants were seated in front of a 22″ monitor with a refreshing rate of 60 Hz, viewed from a distance of 60 cm. Responses were executed on a standard QWERTZ keyboard.

### Data Analyses Strategy

The program IBM SPSS 23 was used for data screening and data analyses. To test our hypotheses, we used model 18 of the macro PROCESS [53], which enables testing intention and EFs as moderators of the proposed mediation effect (Fig. 1). Age and sex are usually correlated with PA behavior [54] and were, therefore, inserted as control variables of the mediator and the dependent variable in the analyses. Furthermore, past PA behavior and planning at t1 were entered to control for baseline values. The factor treatment condition (i.e., treatment group) was used as the independent variable, planning (t2) served as the mediator, and PA intention (t1) and EF measures (t1) were inserted as moderators of the mediation effect, predicting PA behavior at t2. All predictor variables were z-standardized in the case of continuous variables and dummy coded in the case of dichotomous variables (e.g., 0 = control group, 1 = planning group). EF factor scores for inhibition, updating, and shifting were calculated using the respective two tests and the regression method within an exploratory factor analysis. The significance level was set to *p* < .05.

Separate models were tested for each EF. In the case of significant interaction effects, moderation analyses were conducted. Participants were split into groups of higher, average, and lower PA intention, as well as EF performance factor scores. The significance of the regression slopes (simple slopes at the mean as well as at 1 standard deviation (SD) below and above the mean of the intention and EF factor score, respectively), predicting PA behavior from the planning score, was tested for each group separately [53, 55]. The indirect effects at different values of the moderator (moderated mediation effects) were examined using the same intention and EF factor score groups, while testing the significance of the indirect effect for each group separately [53, 55, 56].

## Results

### Descriptive Statistics

With regard to past PA at t1, 25.4% of the participants were not physically active at all, 22.7% were active between 0.5 and 1 h per week, 31.3% between 1.5 and 2 h per week, and 20.6% were physically active for 2.5 h per week or more. Other descriptive statistics and correlations between study variables are depicted in Table 1. As can been seen in this table, past PA, PA intention, and planning are moderately correlated (*r* = 0.35 to *r* = 0.48). Measures of the EFs and EF factor scores were not significantly associated with intention, planning, or past PA behavior at t1 (*r* = − 0.13 to *r* = 0.11).

**Table 1:** Means, standard deviations, miminal scores (min), maximal scores (max), and correlations among study variables (*n* = 176–191)

	1	2	3	4	5	6	7	8	9	10	11	12	13	14	15
1 Age	-														
2 Sex	0.31***	-													
3 PA intention	0.07	0.23**	-												
4 Past PA (t1)	0.04	0.09	0.48***	-											
5 Planning (t1)	0.03	− 0.01	0.45***	0.35***	-										
6 Go/no-go (inhibition)	0.02	− 0.03	0.04	− 0.06	− 0.06	-									
7 Stop-signal (inhibition)	0.01	− 0.04	0.06	0.00	0.05	0.25***	-								
8 N-back (updating)	0.16*	0.27***	− 0.01	0.05	0.01	− 0.13	0.06	-							
9 Visual memory (updating)	0.04	0.18*	0.02	0.12	0.01	− 0.12	0.04	0.39***	-						
10 Alternating runs (shifting)	0.08	0.03	0.07	− 0.02	− 0.13	0.25**	0.03	− 0.10	− 0.12	-					
11 Task-cueing (shifting)	− 0.04	− 0.19	0.08	− 0.03	− 0.09	0.26***	− 0.05	− 0.16*	− 0.07	0.46***	-				
12 Inhibition factor score	0.02	− 0.03	0.06	− 0.05	− 0.01	0.79***	0.79**	− 0.04	− 0.04	0.17*	0.12	-			
13 Updating factor score	0.10	0.27**	0.00	0.11	0.03	− 0.15*	0.04	0.83***	0.83***	− 0.15*	− 0.13	− 0.06	-		
14 Shifting factor score	0.03	− 0.04	0.09	− 0.01	− 0.12	0.30**	− 0.02	− 0.18*	− 0.12	0.86***	0.85***	0.17*	− 0.19*	-	
15 PA (t2)	0.05	0.13	0.45***	0.29***	0.24**	0.15*	0.04	− 0.03	− 0.04	0.01	0.01	0.12	− 0.03	0.01	-
*M*	22.70	71.7%^a^	2.70	1.47	2.17	469.91	258.71	0.68	0.48	643.04	122.74	0.01	− 0.01	0.01	2.91
*SD*	2.53	-	2.01	1.22	0.70	55.57	142.27	0.18	0.26	263.53	100.32	1.00	1.00	0.99	2.19
Min	18	-	0	0	1.00	318.67	50.00	0.00	0.00	− 71.15	− 22.11	− 2.39	− 2.99	− 2.44	0
Max	34	-	8.0	6	4.00	623.45	500.00	1.00	1.00	1423.36	447.82	2.69	2.08	2.84	12

*p < 0.05

**p < 0.01

***p < 0.001

^a^Percentage of women

*PA*, physical activity; *t1*, first assessment; *t2*, second assessment

Inhibition and updating factor scores correlated significantly with shifting, *r* = 0.17, *p* = 0.02, and *r* = −.19, *p* = 0.01, respectively. There was no significant correlation between inhibition and updating (*r* = − 0.06, *p* = 0.43). (Note again that higher updating scores indicate improved performance while higher inhibition and shifting scores indicate impaired performance.)

### Pretest Comparison of Treatment Groups

To determine the comparability of the two treatment groups (planning group vs. control group), independent samples *t* tests were conducted for baseline (t1) study variables (Table 2). The two groups did not differ in any of the assessed variables, and no significant differences were found for sex, *χ*^2^(1, *n* = 191) = 2.43, *p* = 0.12 (planning group *n* = 24 men, *n* = 78 women; control group *n* = 30 men, *n* = 59 women). These findings pointed to the comparability of the two treatment groups with regard to relevant study variables.

**Table 2:** The first assessment (t1) comparison of the treatment groups in terms of relevant study variables

Variable	Statistics						
	*t*	*df*	*p*	*M*_PG_	SD_PG_	*M*_CG_	SD_CG_
Age	0.84	189	0.40	22.59	2.43	22.90	2.71
Past PA	1.41	189	0.16	1.36	1.12	1.61	1.31
PA intention	0.12	189	0.91	2.71	1.97	2.74	2.19
Planning	− 1.02	189	0.31	2.45	0.65	2.35	0.73
Go/no-go	− 0.08	185	0.93	470.02	54.63	469.35	55.61
Stop-signal	1.13	188	0.26	249.29	141.78	272.63	142.28
N-back	0.85	180	0.40	0.67	0.18	0.69	0.18
Visual memory	− 0.29	183	0.77	0.48	0.26	0.47	0.25
Alternating runs	0.24	187	0.81	643.99	265.78	652.92	253.02
Task-cueing	− 1.05	189	0.30	130.62	107.63	115.43	90.68
Inhibition factor score	0.71	184	0.48	− 0.4	1.02	0.07	0.99
Updating factor score	0.16	174	0.87	− 0.02	1.02	0.00	1.00
Shifting factor score	− 0.36	187	0.72	0.03	1.05	− 0.02	0.92

*PG*, planning group; *CG*, control group; *PA*, physical activity

### Testing the Moderated Mediation Models

The treatment condition was a significant predictor of planning at t2. Participants in the planning condition held a higher planning score compared with the control condition. Furthermore, planning at t1 was a positive and significant predictor of planning at t2. With regard to PA behavior at t2, the treatment condition, PA intention, and planning at t2 significantly predicted PA behavior. However, only the interaction effects intention × updating, planning × updating, and planning × intention × updating were significant (Table 3).

**Table 3:** Results of the three moderated mediation models with planning as meditor and intention and inhibition, updating, and shifting factor scores respectively as moderators predicting physical activity behavior (t2)

Moderator	Inhibition (*n* = 184)				Updating (*n* = 174)				Shifting (*n* = 187)			
	*B*	SE	*t*	*R*^2^	*B*	SE	*t*	*R*^2^	*B*	SE	*t*	*R*^2^
Outcome planning (mediator)				0.38***				0.36***				0.38***
Age	− 0.01	0.06	− 0.22		− 0.02	0.06	− 0.24		− 0.00	0.06	− 0.01	
Sex	0.01	0.06	0.12		− 0.00	0.07	− 0.05		− 0.01	0.06	− 0.11	
Past PA (t1)	0.06	0.07	0.96		0.09	0.07	1.25		0.08	0.06	1.20	
Planning (t1)	0.48***	0.06	7.61		0.45***	0.07	6.75		0.48***	0.06	7.61	
Condition (CG vs. PG)	0.62***	0.12	5.17		0.64***	0.12	5.17		0.61***	0.12	5.04	
Outcome physical activity behavior				0.44***				0.47**				0.44***
Age	− 0.02	0.14	− 0.16		0.03	0.14	0.25		0.05	0.14	0.40	
Sex	0.23^+^	0.14	1.66		0.23	0.15	1.55		0.21	0.14	1.51	
Past PA (t1)	0.18	0.16	1.13		0.08	0.16	0.50		0.16	0.15	1.08	
Planning (t1)	− 0.16	0.16	− 0.99		− 0.07	0.17	− 0.39		− 0.25	0.17	− 1.47	
Condition (CG vs. PG)	0.80**	0.28	2.88		0.82**	0.29	2.87		0.70*	0.28	2.48	
PA intention	1.35***	0.25	5.49		1.53***	0.25	6.16		1.32***	0.26	5.16	
Planning (t2)	0.74***	0.17	4.42		0.71***	0.17	4.19		0.82***	0.17	4.71	
EF factor score	0.29*	0.13	2.18		− 0.50	0.14	− 0.36		0.10	0.14	0.69	
Intention × EF factor score	0.03	0.21	0.14		0.49*	0.22	2.21		0.09	0.21	0.42	
Planning × intention	0.12	0.19	0.64		0.32	0.20	1.59		0.24	0.20	1.16	
Planning × EF factor score	0.05	0.14	0.35		− 0.45**	0.15	− 2.93		0.04	0.14	0.30	
Planning × intention × EF factor score	− 0.11	0.20	− 0.55		− 0.45*	0.23	− 2.00		− 0.31^+^	0.18	− 1.76	

^+^*p* < 0.10

*p < 0.05

**p < 0.01

***p < 0.001

*t1*, first assessment; *t2*, second assessment; *PG*, planning group; *CG*, control group; *EF*, executive function; *PA*, physical activity

**Fig. 2:**
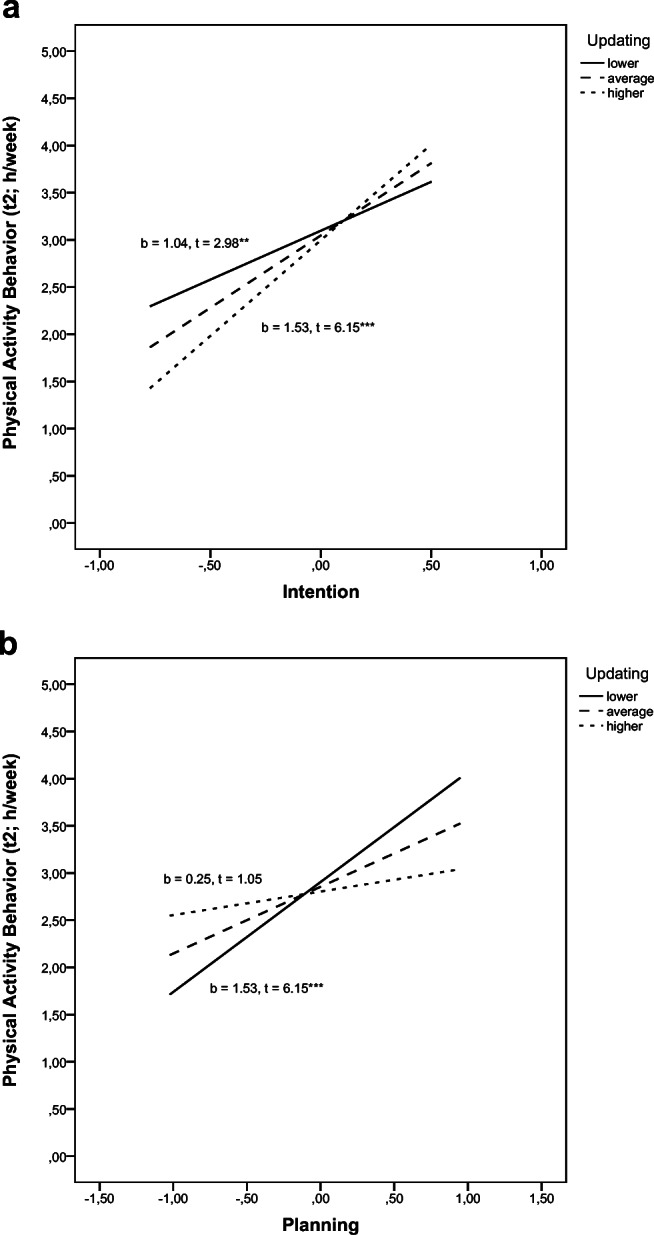
Moderation analyses for the significant interaction effects of intention × updating performance (**a**) and planning × updating performance (**b**) predicting physical activity behavior (t2). t2, second assessment; **p < .01, ***p < .001

Moderation analyses of the significant interaction effect intention × updating revealed that the predictive power of intention for PA (t2) was weakest but significant for those with lower updating factor scores (lower updating performance) (*b* = 1.04, *t* = 2.98, *p* = 0.003) and stronger and significant for those with average updating performance (*b* = 1.53, *t* = 6.15, *p* < .001). For those with higher updating factor scores (higher updating abilities), intention was the strongest significant predictor of the criterion (*b* = 2.03, *t* = 6.30, *p* < .001) (Fig. 2a).

Moderation analyses of the significant interaction effect planning × updating revealed that the predictive power of planning for PA (t2) was strongest and significant for those with lower updating scores (lower updating performance) (*b* = 1.17, *t* = 5.25, *p* < .001) and less strong but still significant for those with average updating performance (*b* = 0.71, *t* = 4.19, *p* < .001). For those with higher updating factor scores (higher updating abilities), planning was not a significant predictor of PA behavior (*b* = 0.25, *t* = 1.05, *p* = 0.294) (Fig. 2b).

**Fig. 3:**
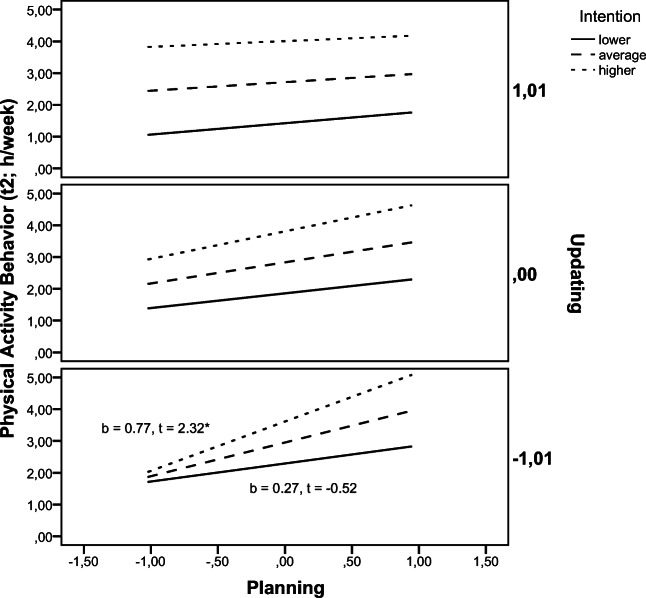
Moderation analyses for the significant interaction effect of planning × intention × updating performance predicting physical activity behavior (t2). t2, second assessment; *p < .05

Moderation analyses of the significant interaction effect planning × intention × updating revealed that the predictive power of planning for PA (t2) was significant when updating factor scores were lower to average and not significant when updating performance was higher. The conditional effect of planning × intention at values of updating factor scores revealed that this effect was significant for those with lower updating factor scores (*b* = 0.77, *t* = 2.32, *p* = 0.022) and not significant for those with average updating scores (*b* = 0.32, *t* = 1.60, *p* = 0.112) and with higher updating scores (*b* = 0.27, *t* = − 0.52, *p* = 0.601) (Fig. 3).

The indices of moderated mediation were not significant for performance in inhibition (*b* = − 0.07, SE = 0.14, 95% CI [− 0.358, 0.211]), updating, (*b* = − 0.29, SE = 0.20, 95% CI [− 0.741, 0.053]), and shifting (*b* = − 0.19, SE = 0.15, 95% CI [− 0.504, 0.091]). The conditional indirect effects of the treatment group on behavior revealed that planning was a significant mediator between treatment group and behavior, irrespective of inhibition performance. However, in the model including updating, planning was a significant mediator when updating performance was lower to average. For participants with higher updating performance, planning was not a significant mediator between the treatment group and behavior. In the shifting model, planning was a significant mediator for all participants except for people with lower intentions and higher shifting abilities.

## Discussion

The aim of the study was to examine how and for whom a short-term planning intervention increases PA behavior. Specifically, the moderating role of intentions and EFs for translating plans into action was investigated.

### Effect of the Planning Intervention on Planning and PA Behavior

As expected in Hypothesis 1, the treatment condition significantly affected planning and PA behavior at t2. Participants in the planning condition showed higher planning scores and more physical activities at t2 compared with participants in the control condition. The current results are in line with previous randomized control trials and meta-analyses documenting the short-term effectiveness of planning interventions for PA behavior promotion when combining action and coping planning [7, 19, 20]. Critically, these effects were relevant prerequisites to further examine if the planning intervention was more successful for people with high intentions and low EFs.

### The Planning–Behavior Association and the Moderating Effect of Intention and EFs

To examine for whom plans translate into action, moderation effects of intention and EFs were examined. The planning score (t2) had a significant influence on PA behavior at t2. Participants that had more detailed plans were more likely to be physically active at t2. The subsequent moderation analysis of the intention × updating interaction revealed that intentions are more likely to be translated into action when updating ability is higher. People with higher updating abilities show stronger intention–behavior relationships (i.e., a smaller intention–behavior gap) compared with participants with lower updating abilities. In a larger context, this result is in line with theoretical assumptions and previous findings [9, 30, 33]. Updating might support the mental representation of a health-related goal and the relevant means by which this goal can be achieved. Updating further enables direct and redirect executive attention to goal-relevant information and, therefore, supports goal shielding, which reduces the intention–behavior gap [30].

The moderation analyses of the planning × updating interaction showed that only for people with lower to average updating performance did planning have a significant influence on behavior enactment. This finding supports our hypothesis that a planning intervention can compensate for low EF abilities. However, according to our study, only lower abilities in updating might be compensated, while inhibition and shifting did not moderate the planning–behavior relationship.

The three-way interaction in the updating model further supported the assumption that planning compensates for poor EF abilities. This is particularly the case when PA intention is high compared with low. For people with high updating abilities, the planning score was not a significant predictor of behavior. Participants with poorer and average abilities in updating benefit from the planning intervention with regard to PA behavior enactment. Participants with higher updating abilities did not benefit from this intervention. In contrast, participants with different levels of shifting and inhibition abilities did not benefit from planning differently.

### The Moderating Role of Intentions and EFs for the Mediation Effect of Planning

Hypothesis 3 (i.e., intentions and EFs moderate the mediation effect of planning) had to be rejected since the indices of moderated mediation were not significant. Conditional indirect effects of the treatment group on behavior at different levels of the moderators (intentions and the EFs) showed that planning was a mediator for most participants. Only for people with high updating abilities was planning consistently not a mediator. Hofmann et al. [30] stated that lower updating abilities might lead to stronger associations between automatic processing and behavior because individuals may follow less effortful courses of action. Individuals with lower updating abilities might benefit from planning because once a situational cue and a behavioral response are linked, the plan is elicited automatically when the situation occurs. Participants with higher updating abilities do not benefit from planning since they might have enough abilities to update their PA goal in daily life and to provide direct attention to goal-relevant stimuli and opportunities, which leads to goal-directed behavior without having prepared plans. They possibly rely on other volitional techniques besides planning, such as spontaneously seizing an opportunity to be physically active [35, 39] in order to realize their PA intentions However, planning was a mediator for participants, almost irrespective of their inhibitory and shifting skills. That is, planning was a strong self-regulatory technique that worked independently of inhibition and shifting performance.

Our results are partly in line with findings of previous studies and extend the findings of Hall et al. [39] and Allan et al. [35], with analyses on single EF tasks to analyses of EFs on a factor level. We found that planning can compensate for poor EF abilities, but, in contrast to Hall et al. [39], we found that updating abilities instead of inhibition were compensated. People with higher EF performance may be better equipped to achieve goals through a better ability to (re-)direct their attention on goal intentions and related information and to remember to act when opportunities arise. Planning may compensate for insufficient levels in executive functioning by letting lower level, automatic, and non-conscious processes determine behavior. As stated above, planning is thought to transfer behavioral control to the environment by mentally linking a situational cue to a specific behavior [10, 57]. As a consequence, this behavior is automatically triggered when the respective cue is encountered. Allowing behavior to be elicited automatically is assumed to circumvent the need for conscious self-regulation via executive functioning [35].

Moreover, Allan and colleagues [35] argue that it is also possible that individuals with strong updating skills use very different techniques to enact intentions that are different from what are implied by planning. For example, it is possible that those with improved updating performance pursue goals by focusing their attention on goal-relevant stimuli and away from tempting stimuli and by keeping their goals in mind during their everyday lives while they look for favorable situations for goal attainment. This technique is quite different from responding automatically to cues to action that heighten accessibility due to repetition of a given behavior in the same situation. Forcing those with strong updating EFs to use an unfamiliar and rigid technique for goal attainment may result in “strategy interference,” where habitual or spontaneous means of self-regulation conflict with the stiff technique provided by planning. In contrast, those with lower EFs may not experience technique interference since they may lack the abilities and techniques for adaptive self-regulation [35, 39]. That is, in the current study, the treatment condition significantly affected the intention–behavior gap (i.e., the planning condition led to a lower intention–behavior gap), even though planning variables are included as mediators in the model. This suggests that further mechanisms explaining the effect of the intervention on the intention–behavior gap exist.

Another possible explanation is given by a theory by Gillebart and de Ridder [58]. Based on their concept of effortless self-control, the authors assume that people with high abilities in self-regulation might be good at automatizing their behavior by forming habits [58]. High self-regulation abilities, such as high trait self-control or strong abilities in updating [30], might help people to establish adaptive and strong PA habits (automatic cue-response associations acquired through context-dependent repetition; [59, 60]) because they do not put themselves in situations where they have to resist temptations [61–63]. Since we included physically inactive as well as physically active participants in our study, it is possible that participants with high updating abilities had already established highly habitualized PA routines. Future research examining the role of EFs in planning intervention studies should distinguish between different phases of health behavior change (e.g., adoption and maintenance as proposed, for example, by the HAPA [8]) and include a measure of behavioral automaticity.

## Limitations

The current study was a randomized controlled study with a 1-week delay between t1 and t2. Our findings represent important results in short-term interventions for PA behavior and the moderating role of EFs. However, it is unclear if these results are transferrable to long-term interventions and PA maintenance. This long-term perspective has to be examined in future studies with longer time intervals, planning booster sessions, and with multiple points of measurements.

Participants in the planning group received intense attention during the planning intervention at t1. In contrast, the control group did not receive the same amount of attention when reading a text. This design might lead to an impact of a social attention effect and to alternative explanations of the present findings. To control for this imbalance in received attention and potential social attention effects, future studies could include a control group with planning intervention regarding an alternative behavior such as eating behavior.

A single-item self-report measure was used to assess PA behavior. Even though this measure has been shown to be sufficiently valid [5, 46–48], this subjective measurement is susceptible for recall bias or social desirability, possibly producing distorted data. Therefore, future studies should use more complex questionnaires to assess PA behavior or, even better, objective measures of PA behavior (e.g., from an accelerometer).

Furthermore, our PA measure was limited to the assessment of high-intensity activities. Since we prompted our participants to plan moderate-to-vigorous physical activities, this raises the question as to whether the results are also valid for activities with lower intensities (light-to-moderate-intensity activities). With regard to behavioral automaticity, some types of activities are more likely to become automatic and habitual than others. More complex and vigorous behaviors are unlikely to be performed completely automatically [64], and more vigorous activities may need more planning than light-intensity activities (such as active-transport walking) since these more vigorous activities require the preparation of sports equipment and sportswear as well as the organization of changing clothes and traveling to sporting venues.

Finally, the study included a sample of healthy undergraduate and graduate students. Therefore, external validity of the results is limited to this specific population and cannot be necessarily transferred to other age groups and educational groups as well as to patients. On the other hand, the present study included a mix of physically inactive as well as physically active participants, making our sample a heterogeneous one from this perspective.

## Conclusions

In sum, planning interventions help people to translate their PA intentions into actions and reduce the intention–behavior gap. The aim of this study was to examine the moderating role of intentions and EFs for the effectiveness of a planning intervention in this domain. In general, high updating abilities supported self-regulation and helped to reduce the intention–behavior gap. Our results showed that planning compensated for poor updating abilities, particularly when intentions were high. Furthermore, the moderated mediation model revealed planning as a mediator of the intervention effect on PA, particularly for those with poor to average updating performance. For individuals with higher updating performance, planning did not represent a relevant technique. Individuals with higher updating performance possibly rely on other self-regulatory techniques than generating and following PA plans. Since action and coping planning represent different planning strategies, future studies should test these two strategies within separate statistical models.

## Footnotes

1 PA, physical activity

2 EFs, executive functions
