# Correction to: Influence of a Planning Intervention on Physical Activity Behavior: The Moderating Role of Intentions and Executive Functions in a Randomized Controlled Trial

**DOI:** 10.1007/s12529-021-09980-2

**Published:** 2021-04-20

**Authors:** Ines Pfeffer, Tilo Strobach

**Affiliations:** Medical School Hamburg, Faculty of Human Science, University of Applied Sciences and Medical University, Am Kaiserkai 1, 20457 Hamburg, Germany

## Correction to: International Journal of Behavioral Medicine (2020) 27:506–519 10.1007/s12529-020-09864-x

The article “Influence of a Planning Intervention on Physical Activity Behavior: the Moderating Role of Intentions and Executive Functions in a Randomized Controlled Trial” written by Ines Pfeffer & Tilo Strobach was originally published Online First without Open Access. After publication in volume 27, issue 5, page 506–519, the author decided to opt for Open Choice and to make the article an Open Access publication. Therefore, the copyright of the article has been changed to ©The Author(s) 2021 and the article is forthwith distributed under the terms of the Creative Commons Attribution 4.0 International License, which permits use, sharing, adaptation, distribution, and reproduction in any medium or format, as long as you give appropriate credit to the original author(s) and the source, provide a link to the Creative Commons license, and indicate if changes were made. The images or other third party material in this article are included in the article’s Creative Commons license, unless indicated otherwise in a credit line to the material. If material is not included in the article’s Creative Commons license and your intended use is not permitted by statutory regulation or exceeds the permitted use, you will need to obtain permission directly from the copyright holder. To view a copy of this license, visit http://creativecommons.org/licenses/by/4.0.

